# Clinical evaluation of an air-capsule technique for the direct measurement of intra-abdominal pressure after elective abdominal surgery

**DOI:** 10.1186/1471-2482-8-18

**Published:** 2008-10-17

**Authors:** Jens Otto, Daniel Kaemmer, Andreas Biermann, Marc Jansen, Rolf Dembinski, Volker Schumpelick, Alexander Schachtrupp

**Affiliations:** 1 Department of Surgery, University Hospital RWTH Aachen, Germany; 2 Department of Anesthesiology, University Hospital of the RWTH Aachen, Germany; 3 Department of Anesthesiology, Karolinen Hospital Arnsberg, Germany

## Abstract

**Background:**

The gold standard for assessment of intraabdominal pressure (IAP) is via intravesicular pressure measurement (IVP). This accepted technique has some inherent problems, e.g. indirectness. Aim of this clinical study was to assess direct IAP measurement using an air-capsule method (ACM) regarding complications risks and agreement with IVP in patients undergoing abdominal surgery.

**Methods:**

A prospective cohort study was performed in 30 patients undergoing elective colonic, hepatic, pancreatic and esophageal resection. For ACM a Probe 3 (Spiegelberg^®^, Germany) was placed on the greater omentum. It was passed through the abdominal wall paralleling routine drainages. To compare ACM with IVP t-testing was performed and mean difference as well as limits of agreement were calculated.

**Results:**

ACM did not lead to complications particularly with regard to organ lesion or surgical site infection. Mean insertion time of ACM was 4.4 days (min-max: 1–5 days). 168 pairwise measurements were made. Mean ACM value was 7.9 ± 2.7 mmHg while mean IVP was 8.4 ± 3.0 mmHg (n.s). Mean difference was 0.4 mmHg ± 2.2 mmHg. Limits of agreement were -4.1 mmHg to 5.1 mmHg.

**Conclusion:**

Using ACM, direct IAP measurement is feasible and uncomplicated. Associated with relatively low pressure ranges (<17 mmHg), results are comparable to bladder pressure measurement.

## Background

Intra-abdominal hypertension (IAH) is defined by a sustained or repeated pathological elevation of intra-abdominal pressure (IAP) to more than 12 mmHg. This condition has been shown to be an independent factor of organ dysfunction and -failure [1-3] and may lead to the abdominal compartment syndrome (ACS) [2]. Both, IAH and ACS have been observed to occur in any patient population needing intensive care with an incidence rate of 50 and 8% respectively [4,5].

Clinical examination of the abdomen in order to detect hypertension has been demonstrated to have an insufficient sensitivity [6]. Therefore IAP measurement has been recommended in patients at risk to develop IAH and ACS [2]. The gold standard for intermittent IAP measurement is the intra-vesicular pressure measurement (IVP) [2]. This measurement principle is widely accepted in the clinical regard [7-9] but has inherent problems with regard to intrinsic bladder wall tension, reference level, body position, discontinuity and indirectness [2,10].

In a porcine model an air-capsule-technique for the direct measurement was applied and this technique showed a high precision and a good agreement with bladder pressure measurement [11]. Although direct intraabdominal pressure measurement is routinely used to validate indirect methods [12-15] it has not been applied for monitoring of patients. It can be argued, that direct intraabdominal pressure measurement is difficult and bears additional risks. However, this has not been examined yet. Aim of the underlying study was to evaluate the air-capsule technique for direct measurement with regard to feasibility and agreement with bladder pressure measurement in patients undergoing abdominal surgery.

## Methods

With approval of the local ethical committee (document-nr. EK-2024) a prospective cohort study was performed between January and August 2003 at the surgical intensive care unit (ICU) of the Department of Surgery, University Hospital of the RWTH Aachen, Germany. The study was conducted in accordance with the study protocol, the Declaration of Helsinki and applicable regulatory requirements.

Included were patients scheduled for elective abdominal surgery: colon resection (35.4%), esophageal resection (18.7%), pancreaticoduodenectomy (12.5%), gastrectomy (10.5%) and liver resection (22.9%). Furthermore, patients were included if an abdominal drain was placed and if postoperative ICU surveillance as well as placement of a Foley-catheter was deemed necessary. The indication for these procedures was based on the standard preoperative assessment and surgical procedure but was not based on study reasons.

Excluded were patients with an age < 18 years, coagulation dysfunction, intraabdominal inflammation, liver insufficiency (Child-Pugh-stage B or C), renal failure with necessity for dialysis and inclusion in other studies.

A total of 30 patients were included (8 female and 22 male) with a mean age of 57.5 years (min. 32 years; max. 75 years). The mean body weight was 75.4 kg with a mean body-mass-index (BMI) of 25.6.

### Study protocol and measurement of IAP

**Figure 1:**
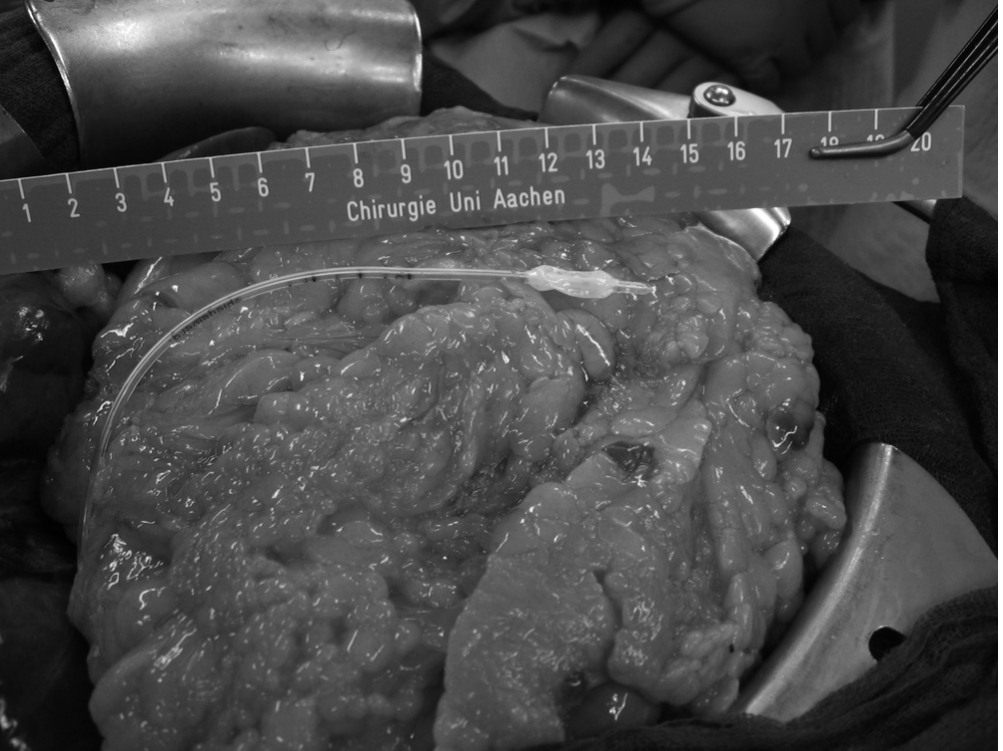
Positioning of the Spiegelberg probe on the greater omentum.

**Figure 2:**
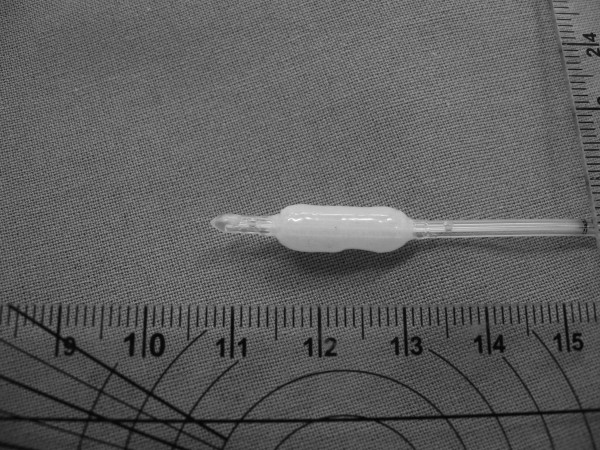
Spiegelberg probe 3; air-capsule system (Spiegelberg, Hamburg, Germany).

**Figure 3:**
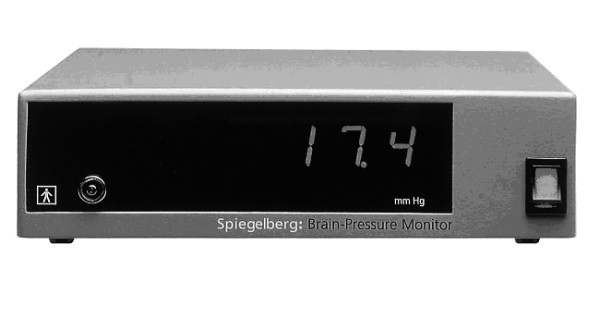
Control and reading device (model HDM 13.3, Spiegelberg, Hamburg, Germany).

For direct IAP measurement, an air-capsule probe (Spiegelberg^®^-System, Probe 3, Hamburg, Germany) was placed during the operation on the greater omentum in midline position cranial of the umbilicus (figure 1). The catheter was then passed through the abdominal wall paralleling the routinely used drains (Easy Flow^®^) and was fixed to the skin with a suture. The system for air-capsule pressure measurement *(ACM) *consisted of the catheter with an air-inflatable capsule situated at the top (outer diameter 2.3 mm, figure 2). The catheter was connected to a control and reading device (model HDM 13.3, Spiegelberg^®^, Hamburg, Germany; figure 3). By maintaining a constant volume in the air-capsule the pressure within the system is made equivalent to the surrounding atmospheric pressure. The system is self-calibrating hourly, does not depend on a reference level and has originally been used for the measurement of the intracranial pressure [11]. Meanwhile, it has also been used for transgastric assessment of IAP [16].

Hydrostatic intravesicular pressure *(IVP) *measurement was done as follows: The tubing system, Foley catheter, and bladder were first flushed with 50 ml sterile saline. This fluid was completely drained leaving no air in situ before another 50 ml saline was injected serving as measurement volume. Using a standpipe, pressure readings were obtained at the end of the exspiration. The level of the symphysis always served as reference and readings in cmH_2_O were converted into mmHg by multiplication with 0.74.

According to a protocol, IVP measurements were done every eight hours, while ACM was performed continuously. In this setting, ACM values were first to be recorded. Then, a reset was performed in order to initiate recalibration of the ACM monitoring system and bladder readings were taken. Simultaneous readings of ACM and IVP were recorded.

ACM value after recalibration was compared to the last ACM value before recalibration and to the corresponding IVP recordings. Comparison of ACM values was performed to assess a possible measurement drift over the period of 8 h. A measurement drift was observed in a previously performed porcine investigation using CO_2 _for the induction of IAH [11].

The intraabdominal catheter was withdrawn whenever the urinary catheter war removed, patients left the intensive care unit (ICU) or after 5 day of ACM measurement. Patients were physically examined twice daily and assessed for dislocation or defect of ACM-catheter as well as for catheter related erosion and infection of adjacent tissue.

### Statistical analysis

After confirming normal distribution of the values with Shapiro-Wilk analysis, results are presented as mean ± SD.

To compare readings derived from IVP and ACM Student's t-test was applied. Also, Pearson's coefficient of correlation (r) was calculated. Lastly, the mean difference and limits of agreement (mean difference ± 1.96 SD) were calculated according to the method of Bland and Altman [17].

## Results

The use of the ACM did not lead to complications and postoperative course of all patients was uneventful. Particularly there were no signs of probe related organ lesion or surgical site infection.

Except for one unintended dislocation of the measurement probe which occurred during a transport of the patient, we registered no catheter malfunction. Furthermore, withdrawal of the measurement probe at the end of the measurement period could be done uneventfully in all patients without any defect of material or complication.

The mean insertion time of the intraabdominal measurement probe was 4.4 days (min-max: 1–5 days). The measurement drift was 0.9 ± 0.8 mmHg. Altogether 168 pairwise measurements of the intraabdominal and the intravesicular pressure were performed.

**Figure 4:**
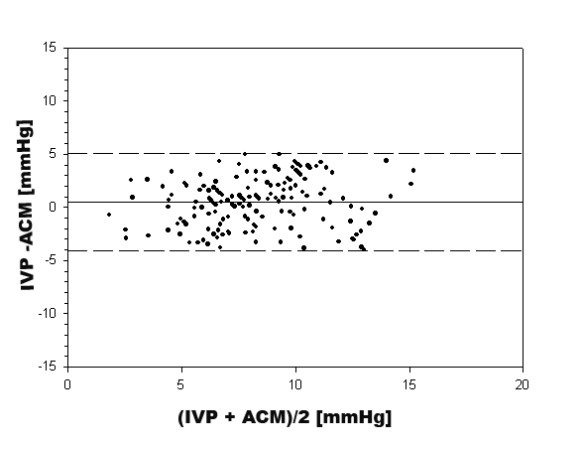
**Pairwise pressure measurements using the intermittent intravesical (IVP) and the intraabdominally placed air-capsule method (ACM).** Difference vs. mean value; 168 measurements in 30 patients.

A mean ACM value of 7.9 ± 2.7 mmHg (min – max: 1.5 – 15.0 mmHg) was recorded while mean IVP reading was 8.4 ± 3.0 mmHg (1.1 – 16.9 mmHg). There was no significant difference (p = 0.29). Pearson's coefficient of correlation was r = 0.69. The mean difference between IVP and ACM was 0.4 mmHg ± 2.2 mmHg. Limits of agreement were -4.1 mmHg to 5.1 mmHg for each device (figure 4).

## Discussion

Direct measurement of IAP has been considered to be invasive [16] but is routinely applied for validation of indirect techniques [12-15]. Brooks recently evaluated a device for a direct and continuous assessment of IAP and reported no complications[18]. Moreover, intraperitoneal measurement – though difficult- has been considered to be needed to fully address the accuracy of IAP measurement in clinical practice [19]. In patients undergoing abdominal surgery for other reasons placement of an intraabdominal measurement probe would be no more hazardous than the placement of abdominal drains [18].

After abdominal surgery, liver transplantation and decompressive laparotomy due to ACS, it has been observed, that IAH occurs often and is an independent risk factor of organ impairment [1,3] while recurrent ACS has an even increased mortality [20]. During the definitive closure of the abdominal cavity after laparostomy, an increase of IAP has been observed [21]. In these patients, direct IAP measurement could help to identify the right time for reconstruction and could also help to monitor postoperative IAP. In addition, direct IAP measurement would be a good alternative in the case that contraindications for intermittent intravesicular pressure measurement are present e.g. local infection, cystic or urethral trauma and cistostomy [22].

Agreement of the air-capsule probe with the intravesicular pressure was 0.4 mmHg with limits of agreement ranging from -4.1 to 5.1 mmHg. This probe has not been validated clinically in the intraabdominal position yet. In a porcine model mean difference to applied pressure was 0.5 with limits of agreement ranging from -4.5 to 5.4 mmHg [11]. In a clinical study, Malbrain et al. placed the Spiegelberg probe intra-gastrically and validated it against laparoscopic pressure measurement. In their study, mean difference to laparoscopic pressure was 0.9 with limits ranging from -0.7 to 2.5 mmHg [14]. It was concluded that ACM is a method of high accuracy and reproducibility and is comparable to intravesical pressure measurement. In a recently published review article, it was stated that a new IAP measurement technique should have a mean difference from -1 to 1 mmHg and limits of agreement within 4 mmHg [19]. Accordingly, the agreement of ACM with IVP in the underlying study was acceptable but less when compared to the aforementioned transgastric use. Furthermore, we have to mention that the good agreement is associated with relatively low pressure ranges (<17 mmHg).

In a previous experimental study, ACM displayed a high measurement to measurement drift which was probably due to a falsified capsule volume caused by the CO_2 _used to increase IAP in that model [11]. In the underlying study, drift was little and unlikely to be the reason for a reduced agreement since values after recalibration were used for comparison of ACM with IVP. A possible cause could be the fact that measurements were performed in two different compartments as already pointed out in other clinical investigations [23,24]. Lastly, absolute values of IAP appear to be less relevant than a reproducible and reliable registration of IAP trend especially in serial measurement [25].

Serial measurement of IAP have been recommended for patients at risk to develop IAH [2]. Intermittent bladder pressure measurement however has been characterized as time- and personnel- consuming [16] which is likely to be the cause for not routinely using it [7]. Although continuous intravesical pressure measurement is available and could be done without extra instruments [25], some issues remain to be investigated: These are the effect of pelvic trauma, detrusor activity and variable bladder compliance [2] but probably more important the influence of reference point and patient positioning. Consequently the latter two issues were subject of a recently completed trial of the world society of the abdominal compartment syndrome (WSACS).

The amount of measurement volume has also been questioned [26]. Malbrain and De Waele investigated the effect of measurement volume on bladder pressure and observed a significant increase with a volume of 25 ml. In the investigation of Malbrain, the increase of IVP only became clinically relevant at a volume of 75 ml for most of the patients and it was concluded that larger instillation volumes than the usually recommended 50 ml to estimate IAP by bladder pressure may cause clinically relevant overestimation of IAP. Kimball recently published a study in which bladder pressure measurement in critically ill patients using 50 ml displayed high reproducibility and reliability [27]. Consequently, the 50 ml used as measurement volume for IVP in the patients of the underlying study appear to be appropriate.

Patients in the underlying study were followed according to the standard postoperative protocols. An increased morbidity due to the use of the intraabdominal measurement probe could not be observed in the patients for a period of up to 5 days. All probes could be easily withdrawn without any complications. A control group without intraabdominal probes was not part of the study as we aimed to basically assess feasibility and agreement with intravesicular pressure measurement. The short observation period and the small number of patients restrict the expressiveness according to direct intraabdominal measurement related complications. Comparable studies are missing but to exlude an additional risk of ACM-catheters further studies are needed.

Another limitation of the underlying study might be that IVP did not exceed 17 mmHg. This was probably caused by the fact that patients were investigated after elective abdominal surgery. Consequently, agreement of ACM with IVP at higher IAP levels cannot be derived. Also, the prognostic value of ACM values – as already known from IVP [1,2,28] – values remains to be confirmed.

## Conclusion

Direct, intraabdominal measurement of IAP was safely performed in 30 patients after elective abdominal surgery for up to 5 days. Beeing aware of relatively low pressure ranges (<17 mmHg), agreement with standard IVP was acceptable in the underlaying study. Direct measurement could be indicated in patients after abdominal surgery who are at risk for the development of IAH e.g. after liver-transplantation, after decompression for ACS and prior to closure of abdominal walls after laparostomy. Although direct intraabdominal pressure measurement appears to be feasible in selected surgical patients, prospective clinical studies are needed to confirm IAP thresholds already known from bladder pressure measurement.

## Competing interests

AS is Scientific Manager for B. Braun Melsungen, Germany which does not manufacture a commercially available kit for intra-abdominal pressure monitoring. The remaining authors have no financial involvement with any organization or entity with a financial interest in or in financial competition with the subject matter or materials discussed in the manuscript.

## Authors' contributions

JO and AS have made substantial contributions to conception and design. DK and MJ have been involved in revising the manuscript critically for important intellectual content. AB and RD have made substantial contributions to acquisation of data. VS have given final approval of the version to be published.

## Pre-publication history

The pre-publication history for this paper can be accessed here:


